# Analytics of the clinical implementation of pharmacogenomics testing in 12 758 individuals

**DOI:** 10.1002/ctm2.586

**Published:** 2021-11-06

**Authors:** Yang Wang, Fan Xiao, Yan Chen, Le‐Dong Xiao, Lei‐Yun Wang, Yan Zhan, Xing‐Liang Xiong, Gang Zhou, Rong Liu, Dong‐Sheng Ouyang, Zhi Li, Howard L McLeod, Wei Zhang, Qing Li, Zhao‐Qian Liu, Hong‐Hao Zhou, Ji‐Ye Yin

**Affiliations:** ^1^ Department of Clinical Pharmacology Xiangya Hospital Central South University Changsha P. R. China; ^2^ Hunan Key Laboratory of Pharmacogenetics Institute of Clinical Pharmacology Central South University Changsha P. R. China; ^3^ Engineering Research Center of Applied Technology of Pharmacogenomics Ministry of Education Changsha P. R. China; ^4^ National Clinical Research Center for Geriatric Disorders Changsha P. R. China; ^5^ Hunan Key Laboratory of Precise Diagnosis and Treatment of Gastrointestinal Tumor Changsha P. R. China; ^6^ Geriatric Oncology Consortium Tampa Florida USA; ^7^ USF Taneja College of Pharmacy Tampa Florida USA

Dear Editor,

Pharmacogenomics (PGx) testing is still not widely accepted in routine medical practice, with one of the major obstacles being the lack of evidence to support its clinical benefit.^1^, ^2^ In the Chinese population, large‐scale comprehensive analytics of clinical PGx testing are still lacking.^3^, ^4^ Thus, we analysed clinical PGx testing for personalised drug treatment in 12,758 Chinese patients at Xiangya Hospital of Central South University, which is one of the first institutions to conduct clinical PGx testing in China. We aimed to evaluate both its clinical and cost benefits (cost‐effectiveness).

Our clinical PGx testing unit was a multidisciplinary organisation integrating both the Institute of Clinical Pharmacology and a molecular testing laboratory (Figure 1A). After evaluation, 25 drug‐gene pairs were selected to be tested (Table 1, Figure S1). A total of 12 758 patients were involved (from 2008 to 2020), and their basic demographic data are summarised in Table S1. They originated from at least 18 provinces, which accounted for most regions of the country (Figure 1B). Figures 1C–E show the composition of all tested genes, drug exposure, and diseases. The top three most commonly tested genes were *CYP2C9*, *VKORC1*, and *CYP2C19*, which were genotyped in 7151, 4106 and 3315 subjects, respectively. Warfarin and clopidogrel accounted for more than 10% of all drugs, and they were the most commonly prescribed to patients after PGx testing.

**FIGURE 1:**
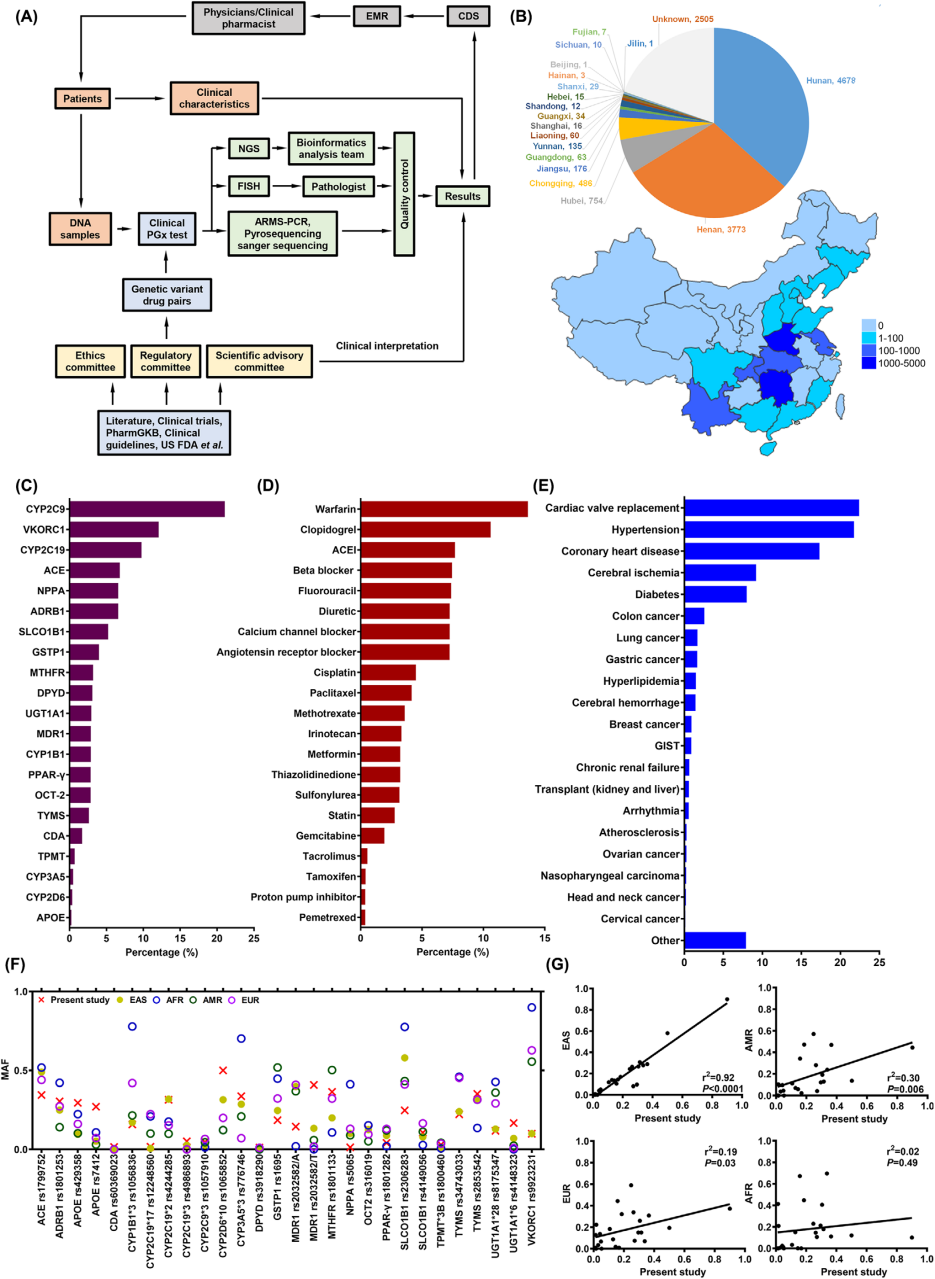
Pharmacogenomics (PGx) testing unit and characteristics of the participants, tested genes, drugs, diseases, and variants. (A) Organisational framework of the clinical PGx testing unit in Xiangya Hospital, Central South University. (B) Geographical distribution of 12 758 participants. The colour and number in the legend indicate the number of participants in different provinces in China. (C–E) Composition of all tested genes (C), drug exposures (D), and diseases (E). They were sorted by the proportion of total number. (F) Comparison of mutation frequencies of tested genes in the present study and East Asian, African, American, and European populations. (G) Correlation analysis of all tested mutations’ MAFs between the present study and East Asian, African, American, and European populations. The frequency data of other populations were retrieved from the dbSNP database. ACEI, angiotensin‐converting enzyme inhibitor; AFR, African; AMR, American; ARMS‐PCR, amplification refractory mutation system‐PCR; CDS, clinical decision support; EAS, East Asian; EMR, electronic medical record; FISH, fluorescence in situ hybridisation; FDA, Food and Drug Administration; GIST, gastrointestinal stromal tumour; MAF, minor allele frequency; NGS, next‐generation sequencing

**TABLE 1:** Drug‐gene pairs and level of evidence

**Drug** *	**Genes** *	**Level of evidence** ^#^	***N* (%)**
Clopidogrel	CYP2C19	A	3,192 (10.89)
Proton pump inhibitor	CYP2C19	A	113 (0.39)
Warfarin	CYP2C9/VKROC1	A	4,106 (14.01)
Tacrolimus	CYP3A5	A	167 (0.57)
Fluorouracil	DPYD	A	1,053 (3.59)
Statins	SLCO1B1	A	817 (2.79)
Irinotecan	UGT1A1	B	113 (0.39)
Angiotensin‐converting enzyme inhibitor	ACE	B	2,316 (7.90)
Methotrexate	MTHFR	B	1,081 (3.69)
Tamoxifen	CYP2D6	B	123 (0.42)
Cisplatin	TPMT	B	5 (0.02)
Fluorouracil	TYMS	B	124 (0.42)
Cisplatin	GSTP1	C	1,352 (4.61)
Statins	APOE	C	85 (0.29)
Beta‐blocker	ADRB1	C	2,241 (7.65)
Gemcitabine	CDA	C	582 (1.99)
Paclitaxel	CYP1B1	C	974 (3.32)
Sulfonylurea	CYP2C9	C	953 (3.25)
Angiotensin receptor blocker	CYP2C9	C	2,181 (7.44)
Paclitaxel	MDR1	C	310 (1.06)
Pemetrexed	MTHFR	C	113 (0.39)
Calcium channel blocker	NPPA	C	2,241 (7.65)
Diuretic	NPPA	C	2,241 (7.65)
Metformin	OCT2	C	970 (3.31)
Thiazolidinedione	PPAR‐γ	C	970 (3.31)

* The drug‐gene variants pairs were determined based on the following considerations: 1. established evidence from published literature, especially in the Chinese population; 2. annotations from FDA, PharmGKB, CPIC and other clinical guidelines (for example, NCCN and ACCP); 3. the existence of functional or tag SNPs for key pharmacogenes; and 4. specific genes or variants under investigating in our institute.

^#^ Level of evidence was important for giving drug dosing adjustment recommendations or risk warnings. They were assigned as three levels: 1. actionable PGx biomarkers: standard therapy should be changed; 2. strong evidence but some discrepancy existed: standard therapy adjustment was recommended; and 3. conflicting evidence: keep standard therapy but pay attention to the drug response or toxicity during treatment.

Next, we analysed the PGx genotyping results. The details of all the tested variants are summarised in Table S2. The comparison of minor allele frequency between our results and the four other major ethnic populations is shown in Figure 1F. There were significant differences among the various populations in terms of nearly all variants. We further calculated the minor allele frequency correlation coefficients between our data and other populations. The mutations only showed good consistency in the East Asian population (Figure 1G). This result indicated that it should to evaluate clinical PGx testing in different populations because of the remarkable ethnic differences.

To implement PGx testing, all genotyping results were integrated with the patients’ clinical parameters to facilitate appropriate clinical decision support.^5^ As indicated in Table S1, 55.64% of the patients harboured at least one pharmacogene variant. They received drug dosing adjustment recommendations or risk warnings. We further analysed the data according to drugs and genes, and their percentages were variable. As shown in Figure 2A, the recommendation rate for antihypertensive drugs was much higher than that for all other drugs. In our study, antihypertensive drugs were composed of four types of drugs, including beta blockers, calcium channel blockers, angiotensin‐converting enzyme inhibitors and angiotensin receptor blockers. They were tested using a panel of four genes, and 76.5% of the patients benefited from PGx testing. This result suggested that simultaneous testing of multiple genes by a panel may improve the clinical benefits. Based on the results sorted by genes, individuals who accepted *CYP2D6* genotyping were most likely to benefit from PGx testing. More than 60% of the patients received the PGx recommendation.

**FIGURE 2:**
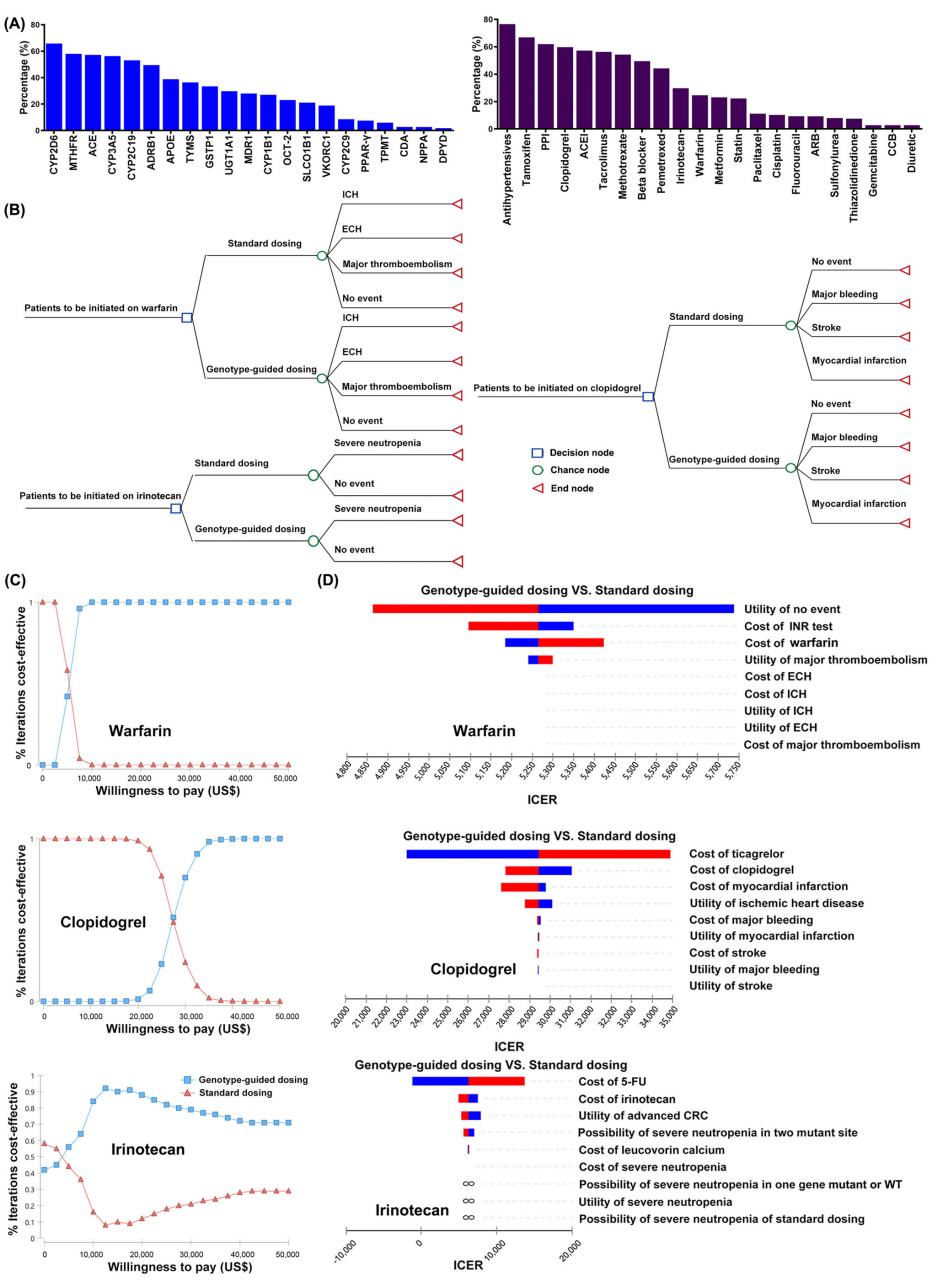
Clinical benefits and cost‐effectiveness evaluation of pharmacogenomics (PGx) testing. (A) PGx recommendations percentage for all tested drugs and genes. All tested drugs and genes were sorted by the recommendation percentage, which was equal to that of the samples that received PGx recommendations (including risk warnings) divided by the total samples tested for each gene or drug. (B–D): Pharmacoeconomics analysis of PGx testing for warfarin, clopidogrel, and irinotecan. (B) Simplified decision tree models of genotype‐guided dosing vs. standard dosing for patients receiving warfarin, clopidogrel, and irinotecan treatments. (C) Cost‐effective curve of warfarin, clopidogrel, and irinotecan under different willingness to pay (WTP) values. The horizontal axis reflects the different WTP values, and the vertical axis represents the cost‐effectiveness possibility of the genotyping‐guided dosing (blue) and standard dosing (red) strategies. (D) Tornado diagrams showing the impact of different variations on the genotyping‐guided dosing versus standard dosing strategies for warfarin, clopidogrel, and irinotecan. The blue and red colours represent decreases and increases in the variable value, respectively. ACEI, angiotensin‐converting enzyme inhibitor, ARB; angiotensin receptor blocker; CCB, calcium channel blockers; CRC, colorectal cancer; ECH, extracranial haemorrhage; ICER, incremental cost‐effectiveness ratio; ICH, intracranial haemorrhage; INR, international normalised ratio; PGx, pharmacogenomics; PPI, proton pump inhibitor

In our study, drug‐gene pairs assigned the ‘A’ level of evidence had actionable PGx biomarkers. The patients were most likely to benefit from testing. Thus, further analysis was conducted in detail. As indicated in Table S3, 6626 patients underwent PGx testing, with a proportion of 51.94% in all individuals. Of these, 76.40% were offered recommendations for drug dosing adjustment. More importantly, 250 patients received PGx clinical decision support alerts, which provided reminders of the increased risk of severe or even fatal clinical events. These results showed that most patients benefit from PGx testing, and some may avoid serious drug toxicity. Multiple‐gene genotyping using a panel is suggested to further improve the benefit rate.

Next, we conducted a pharmacoeconomics analysis to explore the cost‐effectiveness of PGx testing. A decision tree model was used to evaluate the three most tested representative drugs: warfarin, clopidogrel, and irinotecan (Figure 2B). The inputs value and base‐case analysis results are summarised in Tables S4, S5, and Table 2.

**TABLE 2:** Results of the base‐case analysis

**Parameters**	**Warfarin** *		**Clopidogrel** ^#^		**Irinotecan** ^$^	
	**Genotype‐guided dosing**	**Standard dosing**	**Genotype‐guided dosing**	**Standard dosing**	**Genotype‐guided dosing**	**Standard dosing**
Cost per patient per year (US$)	466.8713	385.2739	892.9846	350.9054	19 680.8570	19 567.4206
Incremental cost	81.5974		542.0792		113.4364	
QALY gained per patient year	0.7133	0.6978	0.6567	0.6383	0.7837	0.7656
Incremental QALY	0.0155		0.0184		0.0181	
Incremental cost per QALY gained (US$)	5264.3484		29 460.8261		6267.2044	
Adverse events per patient year	0.0116	0.0224	0.0927	0.1063	0.0402	0.0516
Adverse events averted per patient year	0.0108		0.0136		0.0114	
Incremental cost per adverse event averted (US$)	7555.3148		39 858.7647		9 950.5614	

* For warfarin, the genotype‐guided dosing algorithms and standard dosing strategies were previously described.^9^, ^10^ Adverse events of major thromboembolism, severe intracerebral and extracerebral haemorrhage were the observation endpoints for both arms.

^#^ For clopidogrel, standard dosing patients took clopidogrel at the dose of 75 mg/day without PGx testing. PGx guided dosing patients adjusted medication according to CYP2C19 genotypes as following: wild‐type patients used standard dosing and mutation carriers took ticagrelor 90 mg twice daily.

^$^ For irinotecan, drugs used for standard dosing patients were irinotecan 500 mg/m^2^, calcium leucovorin 200 mg/m^2^ and 5‐FU 400 mg/m^2^. PGx guided dosing patients adjusted medication according to UGT1A1 genotypes. Patients with wild‐type or one‐mutated site of UGT1A1*6 and *28 treated with standard dose, while those with two‐mutated site variants were treated with a 50% dose reduction of irinotecan.

Abbreviation: QALY, quality‐adjusted life‐year.

Incremental outcome (cost, QALY) = Outcome of genotype‐guided dosing – Outcome of standard dosing.

Adverse events per patient‐year = Total adverse events per patient year.

Adverse events per patient‐year averted = Total adverse events of standard dosing – Total adverse events of genotype‐guided dosing.

For warfarin, we evaluated the cost‐effectiveness of *CYP2C9* and *VKORC1* testing during heart valve replacement.^6^ Compared with standard dosing, both the cost of the PGx‐guided dosing group and quality‐adjusted life‐years (QALYs) were higher. The incremental cost‐effectiveness ratio (ICER) was $5264.35 per QALY gained. The willingness to pay (WTP) was notably less, which was $35 661 (Figure 2C). Clopidogrel is used to prevent thromboembolism after percutaneous coronary intervention therapy.^7^ The results showed that the PGx‐guided group increased both the cost ($542.08) and QALYs (0.018). The ICERs were $29 460.83 per QALY gained, which was less than the WTP threshold (Figure 2C). Irinotecan is the first‐line treatment for patients with advanced colorectal cancer, but serious adverse reactions (such as severe neutropenia) may lead to early termination of chemotherapy.^8^ The genotype‐guided group showed increases in both the cost ($113.44) and QALYs (0.018). The ICERs were $6267.20 per QALY gained, which was also much lower than the WTP threshold (Figure 2C). In addition, we performed a sensitivity analysis for these three drugs. Figure 2D shows that the benefit of the ‘genotyping‐guided dosing’ versus that of ‘standard dosing’ medication strategy of warfarin, clopidogrel and irinotecan was mostly affected by the utility of the lack of an event, cost of ticagrelor, and cost of 5‐fluorouracil, respectively. The other parameters in the model had less influence on the ICER. Together, these results showed that our established models are robust, and PGx testing for warfarin, clopidogrel, and irinotecan is cost‐effective.

This study revealed the results of large‐scale clinical PGx testing in the Chinese population for the first time. Our results showed that clinical PGx testing analysis may be recommended in the Chinese population. Most patients may benefit from PGx testing, and multiple‐gene genotyping by a panel may be suggested. However, the coverage of genotyping needs to be improved in the future to provide more comprehensive results. In conclusion, our results provide a reference for patients, physicians, pharmacists, and policymakers in the future for the clinical implementation of PGx testing in both Chinese and other populations.

## CONFLICT OF INTEREST

The authors declare that they have no conflict of interest.

## Supporting information

SUPPORTING INFORMATION

SUPPORTING INFORMATION
