# Supplementary figures and images for: Analytics of the clinical implementation of pharmacogenomics testing in 12 758 individuals
Source: Clin Transl Med. 2021 Nov 6;11(11):e586. doi: 10.1002/ctm2.586 (PMC8571952; doi:10.1002/ctm2.586)

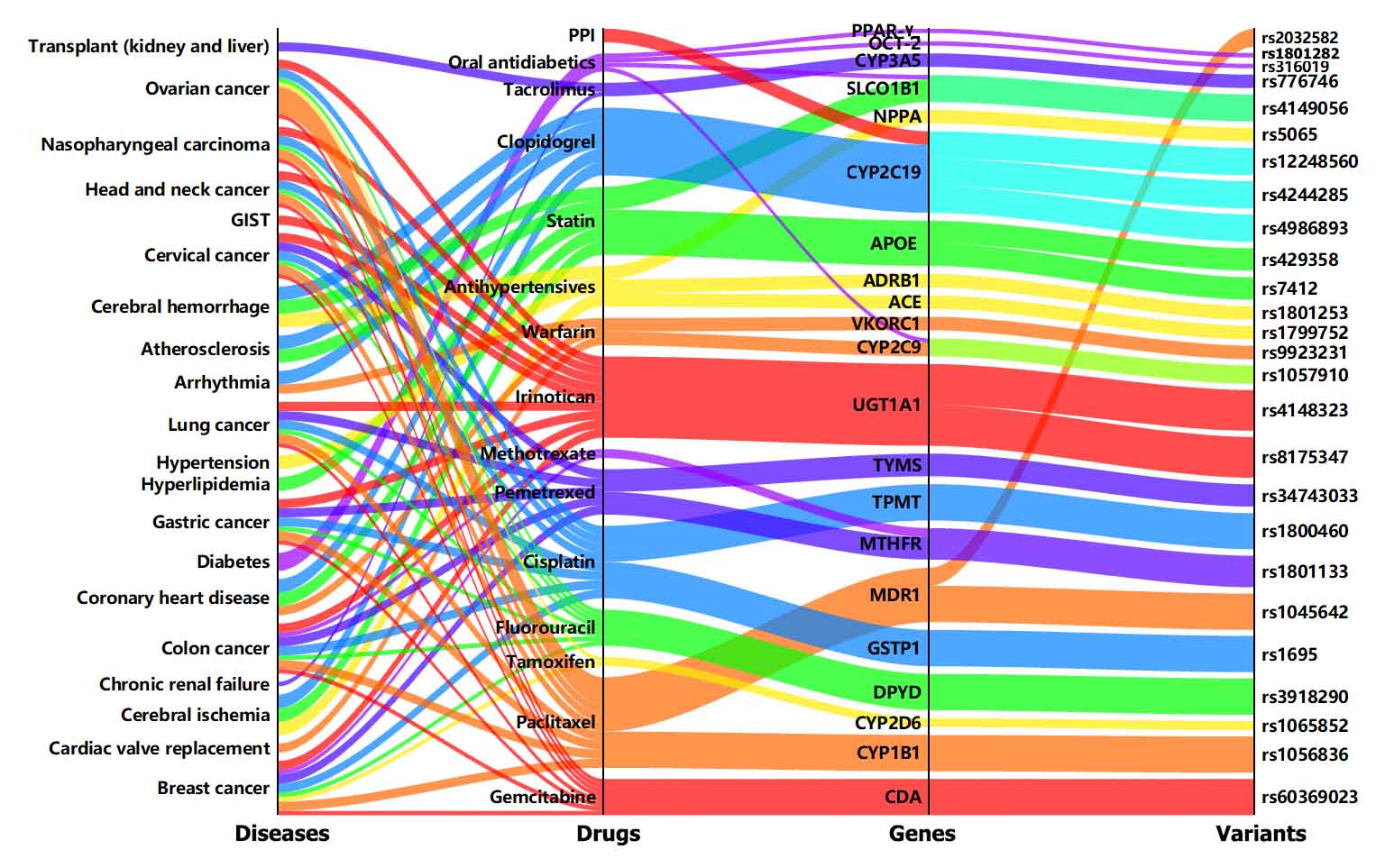

Supplement: Supplementary file 1 — SUPPORTING INFORMATION [file CTM2-11-e586-s001.jpg]
