# Supplementary material for: Analytics of the clinical implementation of pharmacogenomics testing in 12 758 individuals
Source: Clin Transl Med. 2021 Nov 6;11(11):e586. doi: 10.1002/ctm2.586 (PMC8571952; doi:10.1002/ctm2.586)
Supplement: Supplementary file 2 — SUPPORTING INFORMATION [file CTM2-11-e586-s002.docx]

**Analytics of the clinical implementation of pharmacogenomics testing in 12,758 individuals**

Yang Wang^1-3#^, Fan Xiao^1-3#^, Yan Chen^1-3^, Le-Dong Xiao^1-3^, Lei-Yun Wang^1-3^, Yan Zhan^1-3^, Xing-Liang Xiong^1-3^, Gang Zhou^1-3^, Rong Liu^1-3^, Dong-Sheng Ouyang^1-3^, Zhi Li^1-3^, [Howard L McLeod](https://pubmed.ncbi.nlm.nih.gov/?sort=date&term=McLeod+HL&cauthor_id=28549953)^1,5,6^, Wei Zhang^1-3^, Qing Li^1-3^, Hong-Hao Zhou^1-3^, Ji-Ye Yin^1-4*^

^1^Department of Clinical Pharmacology, Xiangya Hospital, Central South University, Changsha 410078; P. R. China; Institute of Clinical Pharmacology, Central South University; Hunan Key Laboratory of Pharmacogenetics, Changsha 410078; P. R. China. ^2^Engineering Research Center of Applied Technology of Pharmacogenomics, Ministry of Education, 110 Xiangya Road, Changsha 410078, P. R. China. ^3^National Clinical Research Center for Geriatric Disorders, 87 Xiangya Road, Changsha 410008, Hunan, P.R. China. ^4^Hunan Key Laboratory of Precise Diagnosis and Treatment of Gastrointestinal Tumor, Changsha 410078, P. R. China. ^5^Geriatric Oncology Consortium, Tampa, FL 33612 USA. ^6^USF Taneja College of Pharmacy, Tampa, FL 33612 USA.

^#^These authors contributed equally to this work.

**Running title**: China clinical pharmacogenomics testing

*To whom correspondence should be addressed: Professor Ji-Ye Yin, Department of Clinical Pharmacology, Xiangya Hospital, Central South University, Changsha 410008; P. R. China. Tel: +86 731 84805380, Fax: +86 731 82354476, E-mail: yinjiye@csu.edu.cn. ORCID: 0000-0002-1244-5045

**Figure Legends for Supplementary Figures.**

**Figure S1:** **Landscape view of the connection of all tested mutations, genes, drugs and diseases.** PPI, proton pump inhibitor; GIST, gastrointestinal stromal tumors.

**Table S1 Demographics of all individuals**

| Characteristics | Parameters |
| --- | --- |
| Number of patients (n) | 12,758 |
| Total recommendations and risk warnings (n, %) | 7,099 (55.64%) |
| Gender |  |
| Male (n, %) | 7,036 (55.15%) |
| Female (n, %) | 5,455 (42.76%) |
| Not reported (n, %) | 267 (2.09%) |
| Age (y, Mean±SD, median) | 56.89±14.24, 57 |
| Weight (kg, Mean±SD, median) | 57.89±10.43, 57 |
| Height (cm, Mean±SD, median) | 160.20±8.13, 160 |
| BMI (Mean±SD, median) | 19.83±2.07, 19.73 |

CDS: clinical decision support, BMI: body mass index

**Table S2: Summary of PGx biomarkers genotyping data**

| Genes | Variants | Methods | Genotypes | N (%) | Assays |
| --- | --- | --- | --- | --- | --- |
| ACE | rs1799752 | Pyrosequencing | DD | 273 (11.79) | LDT |
|  |  |  | ID | 1,053 (45.47) |  |
|  |  |  | II | 990 (42.75) |  |
| ADRB1 | rs1801253 | Pyrosequencing | CC | 1,131 (50.47) | LDT |
|  |  |  | GC | 853 (38.06) |  |
|  |  |  | GG | 257 (11.47) |  |
| APOE | rs429358 | Pyrosequencing | CC | 18 (21.69) | LDT |
|  |  |  | CT | 13 (15.66) |  |
|  |  |  | TT | 52 (62.65) |  |
|  | rs7412 | Pyrosequencing | CC | 55 (66.27) | LDT |
|  |  |  | CT | 11 (13.25) |  |
|  |  |  | TT | 17 (20.48) |  |
| CDA | rs60369023 | Pyrosequencing | AA | 2 (0.34) | LDT |
|  |  |  | GA | 14 (2.41) |  |
|  |  |  | GG | 566 (97.25) |  |
| CYP1B1 | rs1056836 | Pyrosequencing | CC | 710 (72.90) | LDT |
|  | *3 |  | CG | 210 (21.56) |  |
|  |  |  | GG | 54 (5.54) |  |
| CYP2C19 | rs12248560 | ARMS-PCR | CC | 1,166 (97.49) | Commercialized kits |
|  | *17 |  | CT | 29 (2.42) |  |
|  |  |  | TT | 1 (0.08) |  |
|  |  | Pyrosequencing | CC | 90 (95.74) | LDT |
|  |  |  | CT | 4 (4.26) |  |
|  | rs4244285 | ARMS-PCR | CC | 560 (46.82) | Commercialized kits |
|  | *2 |  | CT | 519 (43.39) |  |
|  |  |  | TT | 117 (9.78) |  |
|  |  | Pyrosequencing | CC | 994 (46.93) | LDT |
|  |  |  | CT | 913 (43.11) |  |
|  |  |  | TT | 211 (9.96) |  |
|  | rs4986893 | ARMS-PCR | CC | 1,091 (91.22) | Commercialized kits |
|  | *3 |  | CT | 99 (8.28) |  |
|  |  |  | TT | 6 (0.50) |  |
|  |  | Pyrosequencing | CC | 1,915 (90.33) | LDT |
|  |  |  | CT | 191 (9.01) |  |
|  |  |  | TT | 14 (0.66) |  |
| CYP2C9 | rs1057910 | ARMS-PCR | AA | 206 (91.56) | Commercialized kits |
|  |  |  | AC | 15 (6.67) |  |
|  |  |  | CC | 4 (1.78) |  |
|  |  | Pyrosequencing | AA | 6,334 (91.41) | LDT |
|  |  |  | AC | 570 (8.23) |  |
|  |  |  | CC | 25 (0.36) |  |
| CYP2D6 | rs1065852 | Pyrosequencing | AA | 42 (34.15) | LDT |
|  | *10 |  | GA | 39 (31.71) |  |
|  |  |  | GG | 42 (34.15) |  |
| CYP3A5 | rs776746 | Pyrosequencing | AA | 19 (11.38) | LDT |
|  | *3 |  | GA | 75 (44.91) |  |
|  |  |  | GG | 73 (43.71) |  |
| DPYD | rs3918290 | Pyrosequencing | AA | 5 (0.47) | LDT |
|  |  |  | GA | 14 (1.33) |  |
|  |  |  | GG | 1,034 (98.20) |  |
| GSTP1 | rs1695 | Pyrosequencing | AA | 900 (66.57) | LDT |
|  |  |  | GA | 403 (29.81) |  |
|  |  |  | GG | 49 (3.62) |  |
| MDR1 | rs2032582 | Pyrosequencing | AA | 160 (16.38) | LDT |
|  |  |  | AT | 126 (12.89) |  |
|  |  |  | AC | 349(35.72) |  |
|  |  |  | CC | 199 (20.37) |  |
|  |  |  | CT | 126(12.90) |  |
|  |  |  | TT | 17 (1.74) |  |
| MTHFR | rs1801133 | Pyrosequencing | AA | 162 (14.99) | LDT |
|  |  |  | GA | 465 (43.02) |  |
|  |  |  | GG | 454 (42.00) |  |
| NPPA | rs5065 | Pyrosequencing | TC | 60 (2.68) | LDT |
|  |  |  | TT | 2,181 (97.32) |  |
| OCT2 | rs316019 | Pyrosequencing | GG | 746 (76.91) | LDT |
|  |  |  | GT | 200 (20.62) |  |
|  |  |  | TT | 24 (2.47) |  |
| PPAR-γ | rs1801282 | Pyrosequencing | CC | 7 (0.72) | LDT |
|  |  |  | GC | 66 (6.80) |  |
|  |  |  | GG | 897 (92.47) |  |
| SLCO1B1 | rs2306283 | ARMS-PCR | GA | 17 (42.50) | Commercialized kits |
|  |  |  | GG | 23 (57.50) |  |
|  |  | Pyrosequencing | AA | 64 (8.56) | LDT |
|  |  |  | AG | 294 (39.30) |  |
|  |  |  | GG | 390 (52.14) |  |
|  | rs4149056 | ARMS-PCR | CT | 8(20.00) | Commercialized kits |
|  |  |  | CC | 32(80.00) |  |
|  |  | Pyrosequencing | GG | 36 (2.07) | LDT |
|  |  |  | GA | 320 (18.39) |  |
|  |  |  | AA | 1,384 (79.54) |  |
| TPMT | rs1800460 | Pyrosequencing | AA | 221 (94.04) | LDT |
|  | *3C |  | AG | 14 (5.96) |  |
| TYMS | rs45445694 | Sanger sequencing | 2R2R | 72 (8.07) | LDT |
|  |  |  | 2R3RC | 103 (11.55) |  |
|  |  |  | 2R3RG | 150 (16.82) |  |
|  |  |  | 3RC3RC | 114 (12.78) |  |
|  |  |  | 3RG3RC | 297 (33.30) |  |
|  |  |  | 3RG3RG | 156 (17.49) |  |
| UGT1A1 | rs8175347 | Pyrosequencing | TA6 | 786 (78.68) | LDT |
|  | *28 |  | TA6TA7 | 190 (19.02) |  |
|  |  |  | TA7 | 23 (2.30) |  |
|  | rs4148323 | Pyrosequencing | CC | 702 (70.27) | LDT |
|  | *6 |  | CT | 259 (25.93) |  |
|  |  |  | TT | 38 (3.80) |  |
| VKORC1 | rs9923231 | ARMS-PCR | CC | 10 (2.11) | Commercialized kits |
|  |  |  | CT | 72 (15.16) |  |
|  |  |  | TT | 393 (82.74) |  |
|  |  | Pyrosequencing | CC | 50 (1.38) | LDT |
|  |  |  | CT | 643 (17.69) |  |
|  |  |  | TT | 2,941 (80.93) |  |

LDT: laboratory developed test

**Table S3 Dosing recommendations for actionable PGx biomarkers**

| Drugs | Genes | Genotypes/  Phenotypes | N (%) | Dosing recommendation |
| --- | --- | --- | --- | --- |
| Clopidogrel | CYP2C19 | UM  EM  IM  PM* | 17 (1.36)  464 (37.06)  604 (48.24)  167 (13.34) | Standard dosing  Standard dosing  Alternative drug therapy  Alternative drug therapy |
| [PPI](https://www.pharmgkb.org/chemical/PA450480) (omeprazole, lansoprazole, pantoprazole, dexlansoprazole) | CYP2C19 | UM  EM  IM  PM | 2 (0.67)  147 (49.00)  147 (49.00)  4 (1.33) | Increase dose by 100%  Initiate standard dosing, but consider increasing dose by 50–100%  Initiate standard dosing, but consider 50% reduction in daily dose  Initiate standard dosing, but consider 50% reduction in daily dose |
| [Warfarin](https://www.pharmgkb.org/chemical/PA451363) | CYP2C9/VKROC1 | EM/AA  EM/AG  EM/GG  IM/AA  IM/AG  IM/GG  PM/AA*  PM/AG*  PM/GG* | 2,854 (74.05)  617 (16.01)  55 (1.43)  261 (6.77)  51 (1.32)  2 (0.05)  8 (0.21)  5 (0.13)  1 (0.03) | Calculate dose based on IWPC algorithms  Calculate dose based on IWPC algorithms  Calculate dose based on IWPC algorithms  Calculate dose based on IWPC algorithms  Calculate dose based on IWPC algorithms  Calculate dose based on IWPC algorithms  Consider an alternative agent  Consider an alternative agent  Consider an alternative agent |
| Tacrolimus | CYP3A5 | EM  IM  PM | 73 (43.71)  75 (44.91)  19 (11.38) | Increasing dose by 50–100%  Increasing dose by 50–100%  Standard dosing |
| Fluorouracil | DPYD | EM  IM  PM* | 1034 (98.20)  14 (1.33)  5 (0.47) | Standard dosing  Reducing dose by 25% to 50%  Alternative drug therapy |
| Statin (Simvastatin) | SLCO1B1 rs4149056 | TT  TC  CC* | 413 (46.51)  411 (46.28)  64 (7.21) | Standard dosing  Prescribe a lower dose or consider an alternative statin  Prescribe a lower dose or consider an alternative statin |

IWPC: International Warfarin Pharmacogenetic Consortium.

*These results trigger PGx CDS alert.

**Table S4 Cost and utility inputs**

|  |  | Base-case values | Range for sensitivity analysis | Ref |
| --- | --- | --- | --- | --- |
| warfarin | **Cost input (US$)** |  |  |  |
|  | cost of PGx test | 86.9565 | 0 | * |
|  | cost of ECH | 3,148 | 2,203-  4,092 | ([1](#_ENREF_1)) |
|  | cost of ICH | 4,025 | 2,818-  5,233 | ([1](#_ENREF_1)) |
|  | cost of Major thromboembolism | 19,941 | 15,949.60-  23,924.40 | ([2](#_ENREF_2)) |
|  | cost of warfarin (0.5g) | 0.0725 | 0.0493-0.1110 | * |
|  | cost of INR test | 8.6957 | 7.2539-13.4607 | * |
|  | **Utility input** |  |  |  |
|  | utility of HVR | 0.73 | 0.67-0.79 | ([3](#_ENREF_3)) |
|  | utility of ICH | 0.59 | 0.57-0.61 | ([3](#_ENREF_3)) |
|  | utility of ECH | 0.67 | 0.63-0.71 | ([3](#_ENREF_3)) |
|  | utility of major thromboembolism | 0.39 | 0-0.95 | ([4](#_ENREF_4)) |
| Clopidogrel | **Cost input (US$)** |  |  |  |
|  | cost of PGx test | 130.4347 | 0 | * |
|  | cost of stroke | 66,250 | 32,528-  66,251 | ([5](#_ENREF_5)) |
|  | cost of major bleeding | 38,007 | 26,505-  44,889 | ([5](#_ENREF_5)) |
|  | cost of myocardial infarction | 38,052 | 18,683.39-  38,052.92 | ([5](#_ENREF_5)) |
|  | cost of clopidogrel(75mg) | 0.4713 | 0.3687-0.5739 | * |
|  | cost of ticagrelor(90mg) | 1.0258 | 0.8270-1.2246 | * |
|  | **Utility input** |  |  |  |
|  | utility of ischemic heart disease | 0.7940 | 0.7770-0.8110 | ([5](#_ENREF_5)) |
|  | utility of stroke | 0.7680 | 0.7420-0.7940 | ([5](#_ENREF_5)) |
|  | utility of myocardial infarction | 0.7780 | 0.7580-0.7980 | ([5](#_ENREF_5)) |
|  | utility of major bleeding | 0.5440 | 0.4810-0.6060 | ([5](#_ENREF_5)) |
| Irinotecan | **Cost input (US$)** |  |  |  |
|  | cost of PGx test | 86.9565 | 0 | * |
|  | cost of severe neutropenia | 1,103 | 827-  1,378 | ([6](#_ENREF_6)) |
|  | cost of leucovorin calcium (100mg) | 4.6645 | 1.9217-8.4783 | * |
|  | cost of 5-FU (1mg) | 0.2373 | 0.0325-0.4422 | * |
|  | cost of irinotecan (1mg) | 2.1713 | 1.7730-2.5697 | * |
|  | **Utility input** |  |  |  |
|  | utility of severe neutropenia | 0.42 | 0.25-  0.55 | ([7](#_ENREF_7)) |
|  | utility of advanced CRC | 0.85 | 0.68–  1.00 | ([7](#_ENREF_7)) |

* The value was calculated using our results.

PGx, pharmacogenomics; HVR, heart valve replacement; INR, international normalized ratio; ICH, intracranial hemorrhage ; ECH, extracranial hemorrhage; CRC, colorectal cancer.

**Table S5 Clinical inputs**

|  |  | **Genotype-guided dosing** | **Standard dosing** | Ref |
| --- | --- | --- | --- | --- |
| Warfarin | **Clinical input** |  |  |  |
|  | Possibility of ICH | 0.0018 | 0.0027 | ([8](#_ENREF_8)) |
|  | Possibility of ECH | 0.0009 | 0.0036 | ([8](#_ENREF_8)) |
|  | Possibility of major thromboembolism | 0.0089 | 0.0161 | ([8](#_ENREF_8)) |
| Clopidogrel | **Clinical input** |  |  |  |
|  | Possibility of stroke | 0.0072 | 0.0086 | ([5](#_ENREF_5)) |
|  | Possibility of major bleeding | 0.0273 | 0.0300 | ([5](#_ENREF_5)) |
|  | Possibility of myocardial infarction | 0.0582 | 0.0677 | ([5](#_ENREF_5)) |
| Irinotecan | **Clinical input** |  |  |  |
|  | Possibility of severe neutropenia with full dose without genotype |  | 0.0516 | ([6](#_ENREF_6)) |
|  | Probability of severe neutropenia, wild-type and one-mutated site variants with full dose | 0.0400 |  | ([6](#_ENREF_6)) |
|  | Probability of severe neutropenia, two-mutated site variants with reduced dose | 0.0428 |  | ([6](#_ENREF_6)) |

1. Yang L, Wu J. Cost-effectiveness of rivaroxaban compared with enoxaparin plus warfarin for the treatment of hospitalised acute deep vein thrombosis in China. BMJ Open. 2020;10(7):e038433-e.

2. Chang S-S, Wu J-H, Liu Y, et al. In-hospital direct costs for thromboembolism and bleeding in Chinese patients with atrial fibrillation. Chronic Dis Transl Med. 2018;4(2):127-134.

3. Kim DJ, Kim HS, Oh M, Kim EY, Shin JG. Cost Effectiveness of Genotype-Guided Warfarin Dosing in Patients with Mechanical Heart Valve Replacement Under the Fee-for-Service System. Appl Health Econ Health Policy. 2017;15(5):657-667.

4. You JH, Tsui KK, Wong RS, Cheng G. Potential clinical and economic outcomes of CYP2C9 and VKORC1 genotype-guided dosing in patients starting warfarin therapy. Clin Pharmacol Ther. 2009;86(5):540-547.

5. Jiang M, You JHS. CYP2C19 LOF and GOF-Guided Antiplatelet Therapy in Patients with Acute Coronary Syndrome: A Cost-Effectiveness Analysis. Cardiovasc Drugs Ther. 2017;31(1):39-49.

6. Gu X, Zhang Q, Chu YB, et al. Cost-effectiveness of afatinib, gefitinib, erlotinib and pemetrexed-based chemotherapy as first-line treatments for advanced non-small cell lung cancer in China. Lung Cancer. 2019;127:84-89.

7. Wei X, Cai J, Sun H, et al. Cost–effectiveness analysis of UGT1A1*6/*28 genotyping for preventing FOLFIRI-induced severe neutropenia in Chinese colorectal cancer patients. Pharmacogenomics. 2019;20(4):241-249.

8. Hao Y, Yang J, Zheng X, Hu Y, Yan X, Zhang L. Chinese Patients With Heart Valve Replacement Do Not Benefit From Warfarin Pharmacogenetic Testing on Anticoagulation Outcomes. Ther Drug Monit. 2019;41(6):748-754.
